# Melting Kinetics of Nascent Poly(tetrafluoroethylene) Powder

**DOI:** 10.3390/polym12040791

**Published:** 2020-04-02

**Authors:** Fotis Christakopoulos, Enrico Troisi, Theo A. Tervoort

**Affiliations:** 1 Department of Materials, Soft Materials, ETH Zurich, 8093 Zurich, Switzerland; fotios.christakopoulos@mat.ethz.ch; 2 SABIC Technology and Innovation, 6160 AH Geleen, The Netherlands; Enrico.Troisi@SABIC.com

**Keywords:** poly(tetrafluoroethylene), melting kinetics, isoconversional analysis, superheating, differential scanning calorimetry, nascent polymers, thermal analysis

## Abstract

The melting behavior of nascent poly(tetrafluoroethylene) (PTFE) was investigated by way of differential scanning calorimetry (DSC). It is well known that the melting temperature of nascent PTFE is about $344{\;}^{\circ }$C, but reduces to $327{\;}^{\circ }$C for once molten material. In this study, the melting temperature of nascent PTFE crystals was found to strongly depend on heating rate, decreasing considerably for slow heating rates. In addition, during isothermal experiments in the temperature range of $327{\;}^{\circ }C<T<344{\;}^{\circ }$C, delayed melting of PTFE was observed, with complete melting only occurring after up to several hours. The melting kinetics of nascent PTFE were analyzed by means of the isoconversional methodology, and an apparent activation energy of melting, dependent on the conversion, was determined. The compensation effect was utilized in order to derive the pre-exponential factor of the kinetic model. The numerical reconstruction of the kinetic model was compared with literature models and an Avrami-Erofeev model was identified as best fit of the experimental data. The predictions of the kinetic model were in good agreement with the observed time-dependent melting of nascent PTFE during isothermal and constant heating-rate experiments.

## 1. Introduction

Understanding the melting and crystallization kinetics of semi-crystalline polymers is important for optimizing melt processing techniques, such as injection molding and extrusion. For this reason, the crystallization kinetics of polymers have been studied extensively [1,2,3,4,5]. In contrast, the melting kinetics of polymer crystals have received somewhat less attention. This might partly be due to the relative insensitivity of the melting transition to heating rate as it is observed for most polymers. Recently, isoconversional kinetics have been used for the analysis of the melting kinetics of polymers [5,6,7,8,9,10,11]. Most experimental kinetic studies of polymer melting were performed by a Kissinger analysis of the dependence of the melting-peak temperature on heating rate. Based on the typical chain-folded morphology of polymer crystals, Toda et al. proposed a nucleation model for polymer melting in which melting starts from cylindrical melt nuclei [7,8]. These isoconversional studies on polymer melting were focused on “common” melt-crystallized materials like poly-(ethylene terephthalate), isotactic polypropylene and poly(ϵ-caprolactone).

In contrast to “common” melt-crystallized polymers, nascent polymer powders, also called “virgin” powders, such as as-polymerized ultra-high molecular weight polyethylene (UHMWPE) and poly(tetrafluoroethylene) (PTFE), show pronounced kinetic effects during melting. For these materials, the melting temperature was found to strongly depend on the heating rate, decreasing considerably for slow heating rates [12,13]. In addition, these nascent polymers exhibit an initially high enthalpy of melting (high crystallinity) and melting temperature when compared to the melt-crystallized material, that only occurs during the first melting and is absent in subsequent heating runs of the melt-crystallized material [10,13,14,15,16].

The special nascent (also called “virgin”) morphology can be achieved by polymerization at low temperatures (relatively inactive catalyst surface) or by using single-site catalysts. Under these conditions, the as-polymerized polymer chain is thought to crystallize before it can entangle with its neighbors. The resulting low-entangled polymer also enables solid-state processing of these powders into strong fibers [17,18].

The melting behavior of nascent UHMWPE powder has recently received considerable attention [13,19,20]. It was shown that the elevated first melting peak is due to superheating of the nascent UHMWPE powder [13,14,16]. It was proposed that the anomalous superheating effect, which is not observed in melt-crystallized UHMWPE, was due to the special morphology of the nascent powder, characterized by adjacent-re-entry crystallization, resulting in a high crystallinity and a low degree of entanglement [13]. There have been several attempts to explain this distinct feature of nascent powders. First interpretations for this phenomenon were related to the presence of extended chain crystals in nascent powders [21]. However, in contrast, it was shown that cross-linking the amorphous phase of nascent polyethylene reactor powder before melting, resulted in a much lower melting point contradicting the presence of chain-extended crystals [22]. Similarly it was shown that etching the material with fumic nitric acid, which degrades only the amorphous regions, resulted in a shift of the melting point towards the standard values for melt-crystallized polyethylene [23].

Another polymer that is known to have the specific nascent-powder morphology is PTFE. During polymerisation of as-synthesized “virgin” PTFE, the growing PTFE macromolecular chain crystallizes almost immediately due to its insolubility in the reaction medium. Superheating during the first (initial) melting of nascent PTFE has already been observed, but an analysis of its kinetics does not exist to the best of our knowledge [12,24]. The relatively large temperature window where kinetic effects of melting of nascent PTFE can be observed experimentally, allows the use of a robust isoconversional analysis to evaluate a large calorimetric dataset.

The objective of the current study is, therefore, to evaluate the melting behavior of nascent PTFE powder by differential scanning calorimetry, using isoconversional kinetic analysis, resulting in an apparent activation energy and pre-exponential factor, both as a function of conversion. This is done without the need of any kinetic model used for the analysis of polymer melting beforehand.

## 2. Materials and Methods

### 2.1. Materials

Nascent PTFE powder, Teflon 7AX was supplied by Dolder AG (Basel, Switzerland). For the thermal analysis, non-isothermal differential scanning calorimetry (DSC) was conducted with a heat flux Mettler Toledo 822e (Greifensee, Switzerland). The DSC experiments were performed under nitrogen environment with a flow rate of 20 mL/min. Indium and Zinc standards were used to perform temperature, heat flow and tau-lag calibration. Tau-lag calibration performs two compensations, matching the program temperature to the reference temperature and adjusting for the heating rate [25]. Matlab R2017b and Igor Pro 7 software were employed to perform all numerical fits and analysis. In the present work, 2 mg(±50 μg) of powder material were used for each DSC run. The melting runs were conducted in the temperature range of $150{\;}^{\circ }$C–380 ${}^{\circ }$C, at eighteen different heating rates, from 1.5 ${}^{\circ }$C/min to 10 ${}^{\circ }$C/min with steps of 0.5 ${}^{\circ }$C/min, that fall within range of tau-lag calibration. For each measurement a new sample was used as the first melting behaviour was under investigation unless otherwise indicated. Three replications were conducted for each heating rate in order to improve accuracy. Infrared (IR) spectra were collected in transmission and in attenuated total reflectance (ATR) mode employing a Bruker Vertex 70 (Ettlingen, Germany) on thin melt-compression molded films (50–100 μm).

### 2.2. Kinetic Analysis

Isoconversional analysis is focused on evaluating reactions at the same extent of conversion, α, obtained during different temperature programs. This is done in a model-free manner without assuming a reaction model beforehand [9,26,27,28]. In the case of melting of polymers, investigated by DSC, this can be achieved in two ways. The first consists of isothermal experiments at different temperatures. Another way is by conducting, non-isothermal, constant heating-rate experiments with a different heating program (heating rate, β) between each experiment. Due to the inaccuracy of the isothermal experiments regarding the amount of material that is molten during the heating to the isothermal temperature and the long times needed for those experiments, non-isothermal experiments were conducted. In general, isoconversional kinetic analysis involves evaluating a dependence of $E_{\alpha }$ on conversion or temperature and using this dependence to predict and explore the mechanisms of thermally stimulated processes [29]. The general form of the basic rate equation for a thermally stimulated process can be written as:

(1) $$\left(\frac{d\alpha }{dt}\right)=k\left(T,\alpha \right)f\left(\alpha \right),$$

where α is the extent of conversion, *t* the time, *T* the absolute temperature, f(α) the mathematical function that represents the reaction mechanism and k(T,α) the rate coefficient [30]. The dependence of the rate coefficient on temperature is typically assumed to be an Arrhenius law [31]:

(2) $$k\left(T,\alpha \right)=A\left(\alpha \right)e^{-E(\alpha )/RT}.$$

Here, E(α) is the activation energy, A(α) the pre-exponential factor and *R* is the universal gas constant. The isoconversional principle states that at a constant extent of conversion, the reaction rate is only a function of temperature [5], and so, according to Equation (1):

(3) $${\left[\frac{\partial \ln(d\alpha /dt)}{\partial T^{-1}}\right]}_{\alpha }={\left[\frac{\partial \ln k(\alpha ,T)}{\partial T^{-1}}\right]}_{\alpha }+{\left[\frac{\partial \ln f(\alpha )}{\partial T^{-1}}\right]}_{\alpha }.$$

The subscript α indicates values related to a certain extent of conversion. According to the isoconversional principle, f(α) and A(α) do not depend on temperature, so that according to Equations (1) and (2) at constant α it follows that:

(4) $${\left[\frac{\partial \ln(d\alpha /dt)}{\partial T^{-1}}\right]}_{\alpha }=-E_{a}/R.$$

Using Equation (4), a model-free value of the apparent activation energy is estimated for each degree of conversion, denoted as $E_{\alpha }$. Depending on the nature of the experimental data, a differential or integral isoconversional method can be used. As the current data originates from DSC measurements, the differential isoconversional method of Friedman has been employed [27]. For a given temperature program $T{\left(t\right)}_{i}$, this method requires knowledge of the reaction rate ${\left(d\alpha /dt\right)}_{i}$ as a function of time and temperature. Integration of the experimental conversion rate then gives the conversion as a function of time, $\alpha {\left(t\right)}_{i}$ for the given $T{\left(t\right)}_{i}$.

Equations (1) and (2) lead to:

(5) $$\left(\frac{d\alpha }{dt}\right)=A\left(\alpha \right)f\left(\alpha \right)e^{-E(\alpha )/RT}$$

and by using the isoconversional method at a constant extent of conversion, α it then follows that:

(6) $$\ln{\left(da/dt\right)}_{\alpha }=\ln\left[A_{\alpha }f\left(\alpha \right)\right]-E_{\alpha }/RT_{\alpha },$$

where $\ln[A_{\alpha }f\left(\alpha \right)]$ is constant. The apparent activation energy $E_{\alpha }$ can be determined by a plot of the left hand side of Equation (6) against the reciprocal absolute temperature for each extent of conversion obtained from the various heating programs. If these plots give straight lines, the functional form of Equation (1) applies and $E_{\alpha }$ can calculated from the slope of the resulting Arrhenius-like plots. In order to be able to reproduce the exact form of the rate equation Equation (1), two more kinetic parameters are missing, the pre-exponential factor (A(α)) and the mathematical function that represents the reaction mechanism (f(α)). In case the apparent activation energy $E_{\alpha }$ is not constant, but dependent on conversion, it has been found that the same experimental curve can be described by several reaction models [32]. This typically happens for complex, multi-step, reactions, where the pre-exponential factor might also change with extent of conversion or temperature. In this case, the reaction mechanism (f(α)) cannot be identified by model fitting directly, but it has been shown that a dependance of the pre-exponential factor on $E_{\alpha }$ can be calculated by employing the compensation parameters [32,33]. The compensation effect states that for model $f_{j}\left(\alpha \right)$, the Arrhenius parameters $\ln A_{j}$ and $E_{j}$, estimated by a single-heating-rate method, are related in the form of the linear relationship of Equation (7). By knowing the activation energy and the pre-exponential factor, the reaction model can be numerically reconstructed from Equation (8) for each temperature program $T{\left(t\right)}_{i}$. Then by comparing with the reaction mechanism models of the common reactions operating in solid state transformations, the physical meaning of the experimental reaction mechanism can be explored.

(7) $$\ln A_{j}=aE_{j}+b,$$

(8) $$f_{i}\left(\alpha \right)={\left(\frac{d\alpha }{dt}\right)}_{\alpha ,i}\left[\frac{\exp(E_{\alpha }/RT{\left(t\right)}_{\alpha ,i})}{A_{\alpha }}\right].$$

The method of estimating the pre-exponential factor through the compensation effect is thus given by the following steps. First, $E_{\alpha }$ is calculated as a function of conversion using the isoconversional method. Then a single-heating rate method is applied in order to calculate pairs of $\ln A_{j}$ and $E_{j}$ using Equation (9), by plotting the left hand side against the reciprocal absolute temperature and through a linear fit by calculating $\ln A_{j}$ and $E_{j}$ from the slope and the intercept [9,32]. For $f_{j}\left(\alpha \right)$ the values from the different literature models are used (Table 1) [34]. That means that for one heating program ($T{\left(t\right)}_{i}$) there will be exactly as many pairs as the models used. In order to improve accuracy in the present work, only the five models that yield the best linear fit for each heating program are used for the rest of the analysis.

(9) $$\ln\left(\frac{1}{f_{j}\left(\alpha \right)}\frac{d\alpha }{dt}\right)=\ln A_{j}-\frac{E_{j}}{RT{\left(t\right)}_{i}}$$

Next, the pairs of $\ln A_{j}$ and $E_{j}$ are fitted to Equation (7) and the parameters *a* and *b* are calculated. Finally, the values of $E_{\alpha }$, calculated from an isoconversional method, are substituted to Equation (7) and the pre-exponential factor is calculated. If there is no variation of $E_{\alpha }$ with conversion, then the pre-exponential factor is also invariant. It was suggested and recently confirmed by Sbirrazzuoli et al. that the compensation effect yields accurate values for the pre-exponential factor, even for multiple reactions with a variable activation energy [32].

## 3. Results and Discussion

### 3.1. Material Characterization

**Figure 1:**
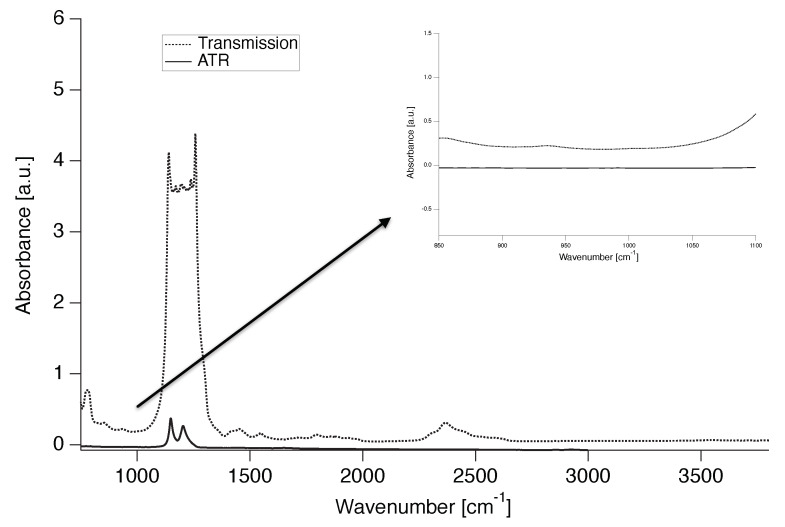
IR spectra of PTFE 7AX in transmission and ATR. Inlet: Magnification of the area of interest (995 cm${}^{-1}$).

Because the PTFE used is a commercial grade, it was tested, using Fourier transform infrared spectroscopy (FTIR) both in transmission and in attenuated total reflection (ATR), for the presence of commonly used co-monomers such as fluorinated propene and perfluorinated alkoxy comonomer. The absence of an absorbance peak at 995 cm${}^{-1}$ indicates no co-monomer content in the PTFE powder (Figure 1) confirming that the PTFE grades used are indeed linear.

**Figure 2:**
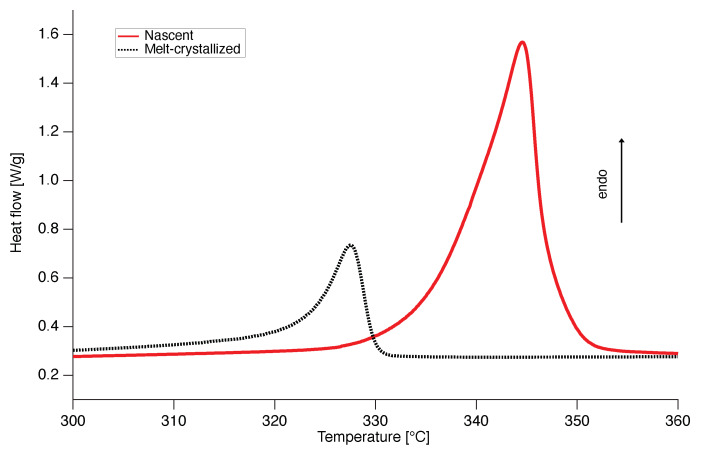
Differential scanning calorimetry thermographs of PTFE 7AX, where the difference in the melting peak temperature and enthalpy between the nascent (red line) and melt-crystallized (black dotted line) is evident.

The high melting peak observed upon melting of nascent PTFE is clearly depicted in the DSC curves of Figure 2, showing the melting temperature of the nascent material at about 345 ${}^{\circ }$C, which is 18 ${}^{\circ }$C higher compared to the melting temperature of the melt-crystallized material at about 327 ${}^{\circ }$C. This first high melting temperature of the nascent material at 345 ${}^{\circ }$C will be referred to as first apparent melting temperature for the rest of the article in order to be distinguished from the melting temperature at 327 ${}^{\circ }$C of the melt-crystallized material. Using the value of $\Delta H_{f}^{0}=82$ J/g for 100% crystalline material [35], it follows from Figure 2 that nascent PTFE has a high crystallinity of 81 wt.%, whereas melt-crystallized has a much lower crystallinity of 32 wt.%, as was found before [12,15].

**Figure 3:**
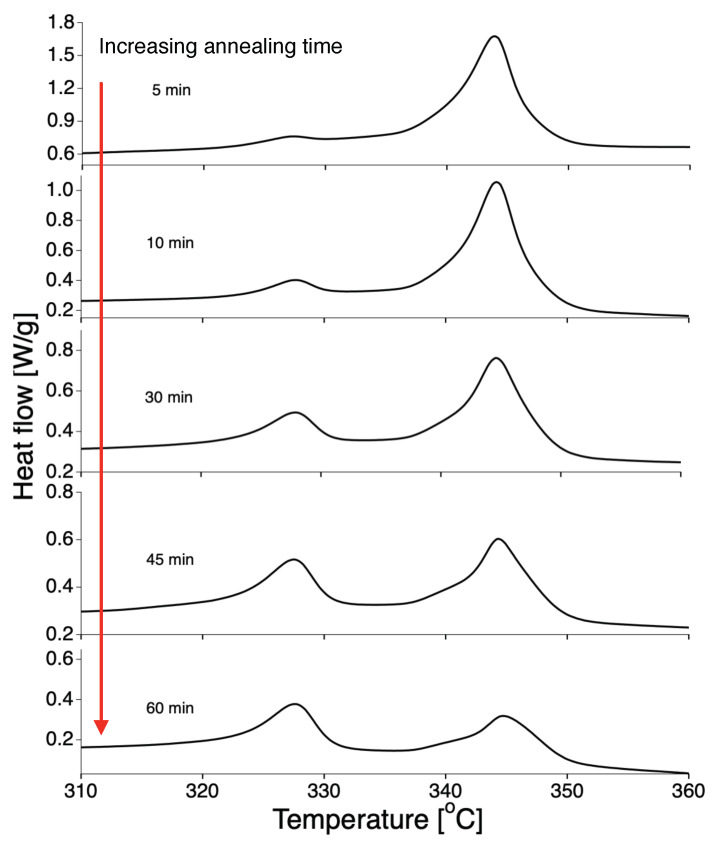
Two-peak melting behaviour indicative of slow melting of nascent PTFE. The DSC thermographs were recorded from room temperature to $360{\;}^{\circ }$C at $10{\;}^{\circ }$C/min. The samples were nascent PTFE, annealed for different times at $335{\;}^{\circ }$C, which is *below* the (apparent) standard melting temperature of nascent PTFE of $344{\;}^{\circ }$C. The first peak at $327{\;}^{\circ }$C relates to melt-crystallized PTFE and increases with annealing time from top to bottom. The loss of crystallinity as a function of time for different annealing temperatures is presented in Figure 12.

It was suggested by Rastogi et al., that nascent reactor powders of UHMWPE exhibit slow melting when annealed below the melting temperature [13]. This slow melting, indicative of slow melting kinetics, can also be observed in nascent PTFE powder. In a typical experiment that demonstrates this slow melting behavior, nascent material is annealed inside the DSC at $335{\;}^{\circ }$C, below the first apparent melting temperature, subsequently cooled down to room temperature and then reheated to $380{\;}^{\circ }$C. The DSC curves of the second heating are presented in Figure 3. The first peak at $327{\;}^{\circ }$C corresponds to the fraction of molten material that crystallized during cooling and upon subsequent heating depicts the typical melting temperature of melt-crystallized material. The second melting peak at $345{\;}^{\circ }$C corresponds to the fraction of nascent material that still has not molten yet.

From Figure 3 it can be observed that even after one hour of annealing the sample at 335 ${}^{\circ }$C, a high melting peak can be observed, indicating that some of the nascent PTFE was still not molten during the annealing step. This cannot be due to poor heat transfer in the DSC pan, but is indicative of extremely slow melting kinetics (superheating). Another way of checking for superheating was suggested by Toda et al. [7], who examined the heating-rate dependence of the melting peak using Equation (10).

(10) $$T_{p,\beta }=T_{m}+B\beta^{z}.$$

**Figure 4:**
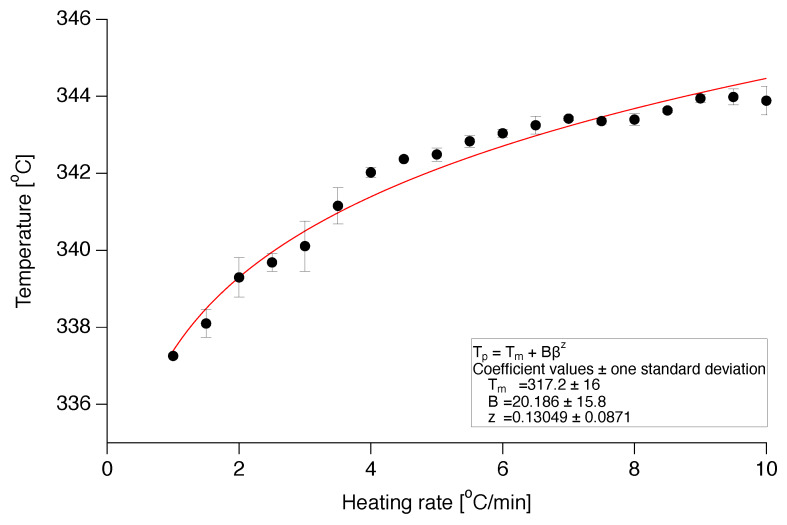
Heating rate dependence of the melting peak temperature of nascent poly(tetrafluoroethylene) (PTFE) crystals.

Here, $T_{p,\beta }$ is the melting peak temperature for each heating rate, $T_{m}$ the non-equilibrium melting temperature and *B* and *z* are fitting parameters. The value of *z* shows the heating rate dependence of the shift in peak temperature and if it is substantially smaller than 0.5, melting can be regarded as kinetically controlled. In the case of nascent PTFE the fitting was performed by having $T_{m}$, *B* and *z* as fit coefficients, since common methods to calculate $T_{m}$, like the Iller’s equation [36], return unrealistic high values that are not in accordance with the experimental observations, possibly due to the special nature of nascent PTFE crystals. From the fitting of $T_{p}$ against β, a value of about 0.13 is obtained for parameter *z* (Figure 4), indicating kinetically controlled melting, in agreement with the isothermal experiments.

### 3.2. Apparent Activation Energy Calculation

**Figure 5:**
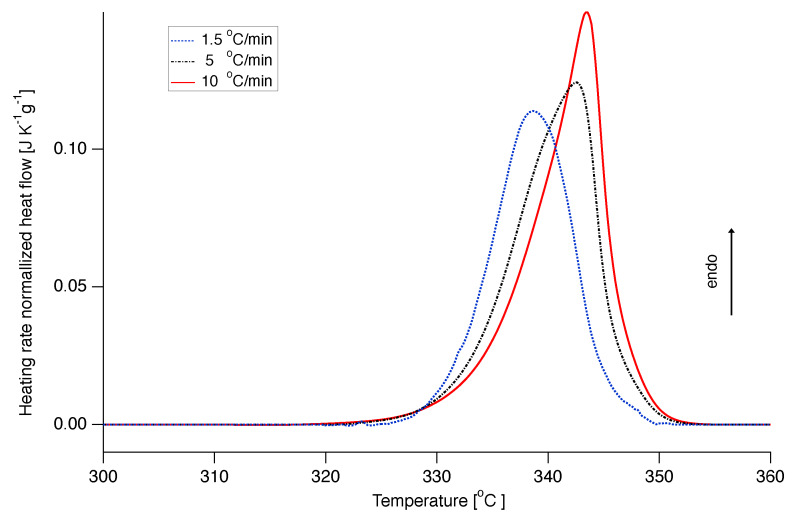
Differential scanning calorimetry thermograhps of nascent PTFE 7AX melting at different heating rates.

Figure 5 demonstrates some DSC curves at different heating rates, with the baseline subtracted and normalized by the heating rate, representative of our melting data.

The differential isoconversional method of Friedman was used for the calculation of the apparent activation energy ($E_{\alpha }$), as the DSC data is already in differential form.

**Figure 6:**
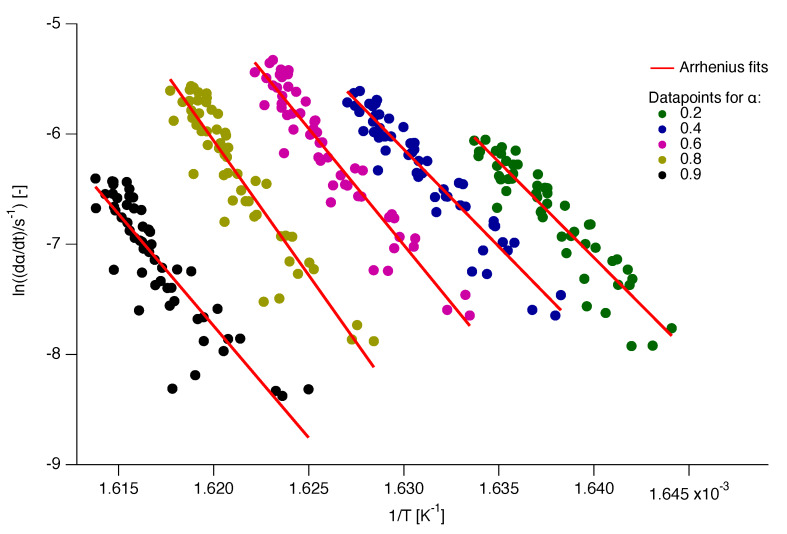
A few representative Arrhenius plots for the determination of the kinetic parameters $E_{\alpha }$ and $A_{\alpha }$.

Some of the Arrhenius plots from which the $E_{\alpha }$ values are calculated are presented in Figure 6. It should be noted that the plots presented in this figure are just a fraction of the data analysed for the $E_{\alpha }$ calculation.

**Figure 7:**
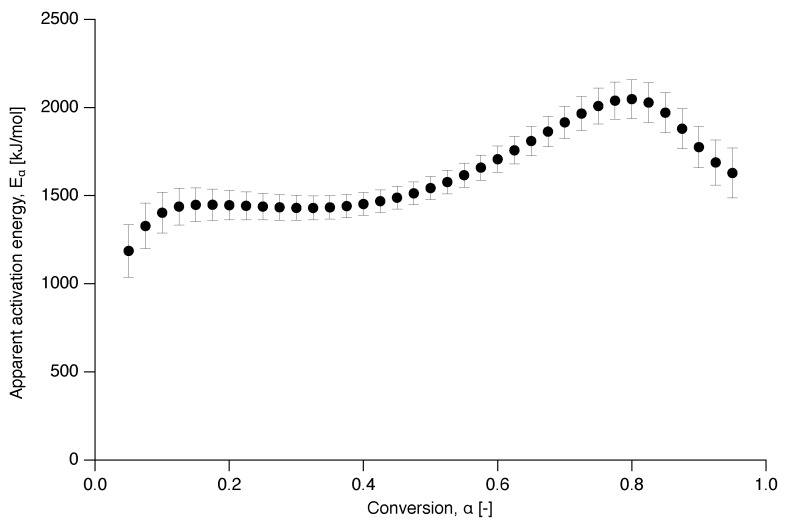
Apparent activation energy as a function of conversion for the nascent PTFE 7AX.

The apparent activation energy dependence on conversion is presented in Figure 7 and shows two distinct behaviors, independent of conversion at the beginning and increasingly dependent after α=0.5 up to α=0.8. This is in contrast to a decreasing apparent activation energy as a function of conversion, as was observed for the delayed meting of certain low-molecular weight materials, such as glucose and fructose [37].

### 3.3. Pre-Exponential Factor and Reaction Model Calculation

**Figure 8:**
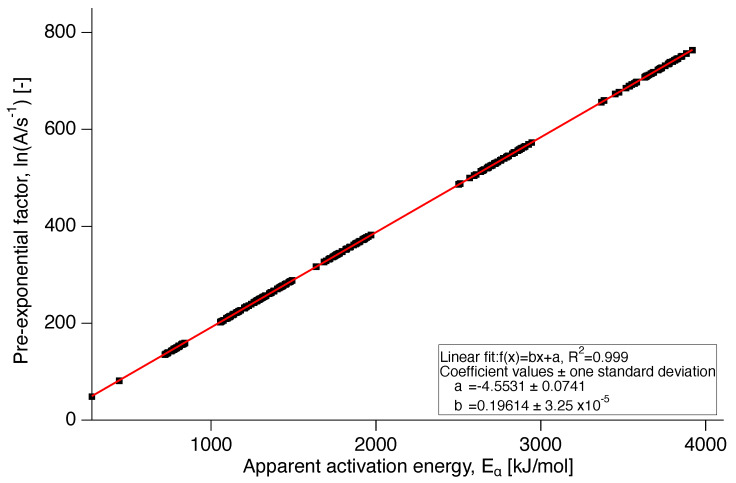
Compensation line for PTFE 7AX.

**Figure 9:**
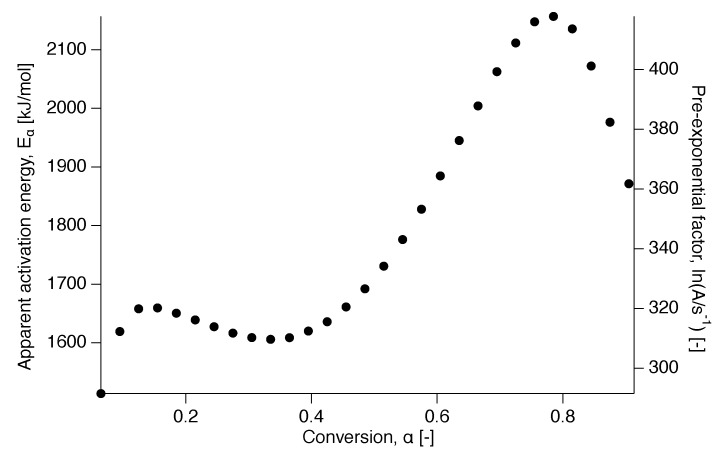
$\ln(A_{\alpha })$ dependencies for PTFE 7AX obtained by the compensation effect.

For the evaluation of the reaction model, the pre-exponential factor was calculated by application of the compensation effect. The compensation line is presented in Figure 8 showing the linear dependence of the natural logarithm of the pre-exponential factor, lnA, on the apparent activation energy in Figure 9. As the apparent activation energy varies with conversion, this is indirectly true for the pre-exponential factor as well through its dependence on $E_{\alpha }$ [9].

**Figure 10:**
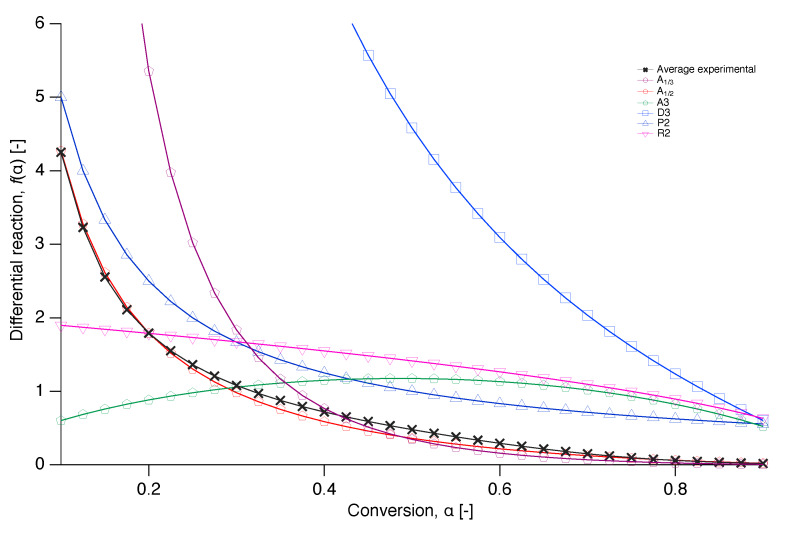
Plots of the differential reaction against the conversion for selected models together with the numerically reconstructed differential reaction of the average value at each conversion of the experimental data.

In order to get an insight into the reaction mechanism of nascent PTFE melting, the differential reaction (f(α)) was numerically reconstructed from Equation (7) and compared with a selection of the models from literature of Table 1 in Figure 10 [34]. A plot where more literature models are included in the comparison can be found in the Appendix A.

**Figure 11:**
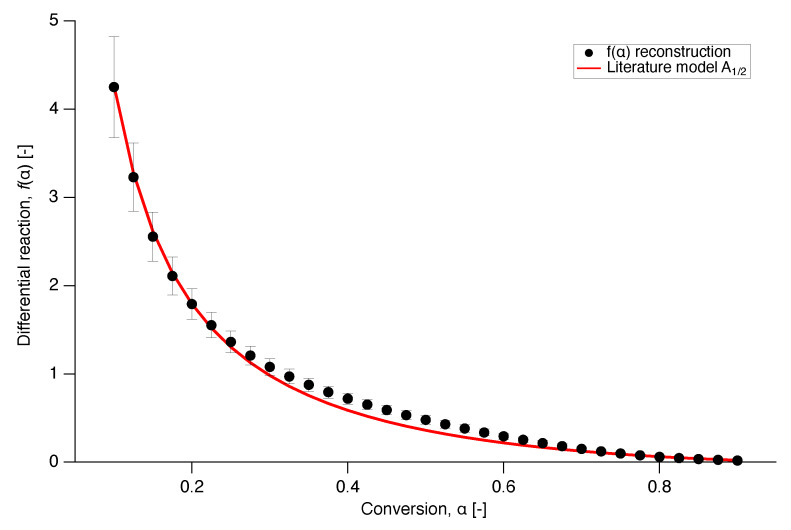
Comparison of the literature model of random nucleation and growth Avrami-Erofeev ${}^{1/2}$ with the experimental data for nascent PTFE 7AX.

The model that provides the best fit to the experimental data is presented in Figure 11, where the differential reaction of literature model $A_{1/2}$ is plotted over conversion together with the numerical reconstruction of the experimental data from all the different heating runs. Model $A_{1/2}$ corresponds to an Avrami-Erofeev random nucleation and growth model with n=0.5.

**Figure 12:**
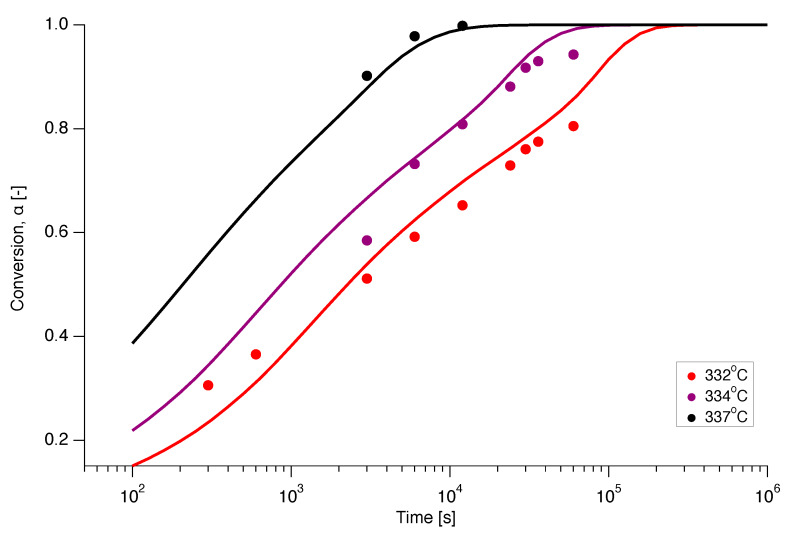
Prediction of the isothermal melting at 332 ${}^{\circ }$C (red), 334 ${}^{\circ }$C (magenta) and 337 ${}^{\circ }$C (black), using the $A_{1/2}$ model (solid line) with the experimentally determined functions for $E_{\alpha }$ and $A_{\alpha }$, compared to the experimental data (dots).

Isothermal experiments were also conducted on nascent PTFE by annealing the sample below the first apparent melting temperature at various temperatures in the range of $332{\;}^{\circ }$C to $337{\;}^{\circ }$C for different annealing times from 5 min up to 1000 min. The dependence of the conversion (melting) on annealing time is presented in Figure 12 for isothermal melting at 332 ${}^{\circ }$C, 334 ${}^{\circ }$C and 337 ${}^{\circ }$C. Ideally, for a clean interpretation of isothermal experiments, no melting should occur during ramping up to the temperature of interest. However, Figure 12 shows that even for the lowest temperature and the the smallest annealing time already 30% of the material is molten. Figure 12 thus illustrates the principal difficulty of obtaining kinetic data for small times for superheating of PTFE through isothermal experiments, especially at higher temperatures.

Figure 12 also shows the prediction of the isothermal melting at 332 ${}^{\circ }$C, 334 ${}^{\circ }$C and 337 ${}^{\circ }$C as a function of time. This was obtained by numerical integration of f(α) of the $A_{1/2}$ model using interpolation functions of the experimentally determined $E_{\alpha }$ and $A_{\alpha }$ relations. Gratifyingly, there appears to be a good correspondence between experiment and prediction.

Interestingly, the isothermal data could also be fitted with the $A_{1/3}$ model corresponding to an Avrami-Erofeev random nucleation and growth model with n=1/3. The integral formulation of the $A_{1/3}$ model is given as:

(11a) $$\begin{array}{cc} \alpha (t) & =1-\exp\left[-{\left(kt\right)}^{1/3}\right] \end{array}$$

(11b) $$\begin{array}{cc} k & =A\exp\left(\frac{-E}{RT}\right). \end{array}$$

**Figure 13:**
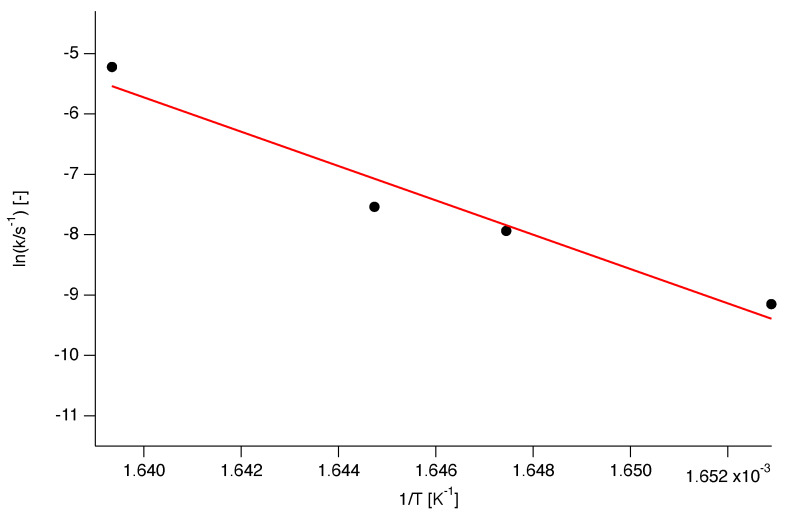
Arrhenius plot of the logarithm of the rate constants ln(k) resulting from the fit of the $A_{1/3}$ model to the isothermal data in Figure 14. The linear fit results in an activation energy of $E=2.36\pm 0.3\times 10^{6}$ J/mol and a logarithm of the pre-exponential factor of $\ln\left(A/s{}^{-1}\right)=4.6\pm 0.6\times 10^{2}$.

The isothermal data obtained at each annealing temperature were fitted with Equation (11a). The Arrhenius plot of the natural logarithm of the rate constants resulting from these fits against the reciprocal absolute temperature, is depicted in Figure 13. From the Arrhenius fit an activation energy of $E=2.4\pm 0.4\times 10^{6}$ J/mol and a logarithm of the pre-exponential factor of $\ln\left(A/s{}^{-1}\right)=4.6\pm 0.7\times 10^{2}$ could be determined. It should be noted that these values for *E* and ln(A) are independent of conversion. It should also be noted that the values of *E* and *A* lie on the compensation line (Figure 8) and that the value of *E* corresponds well with the maximum value of $E_{\alpha }$ as shown in Figure 7.

**Figure 14:**
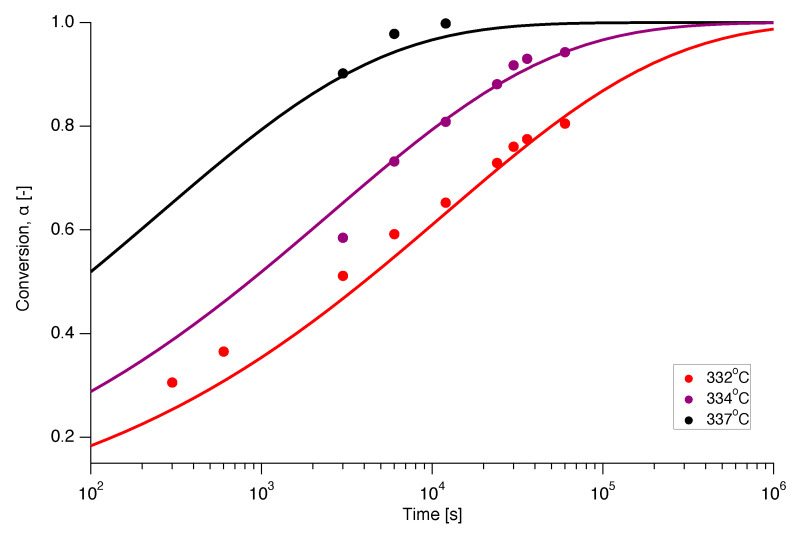
Prediction of the isothermal melting at 332 ${}^{\circ }$C (red), 334 ${}^{\circ }$C (magenta) and 337 ${}^{\circ }$C (black), using the $A_{1/3}$ model (solid line) with constant kinetic parameters, compared to the experimental data (dots).

Using the integral formulation of the $A_{1/3}$ model, Equation (11a) and Equation (11b), together with the kinetic parameters from the Arrhenius fit, allowed for the reconstruction of the isothermal experiments. The resulting curves are shown in Figure 14. As can been seen in this figure, the $A_{1/3}$ model with *constant* kinetic parameters is also in good agreement with the experimental values.

**Figure 15:**
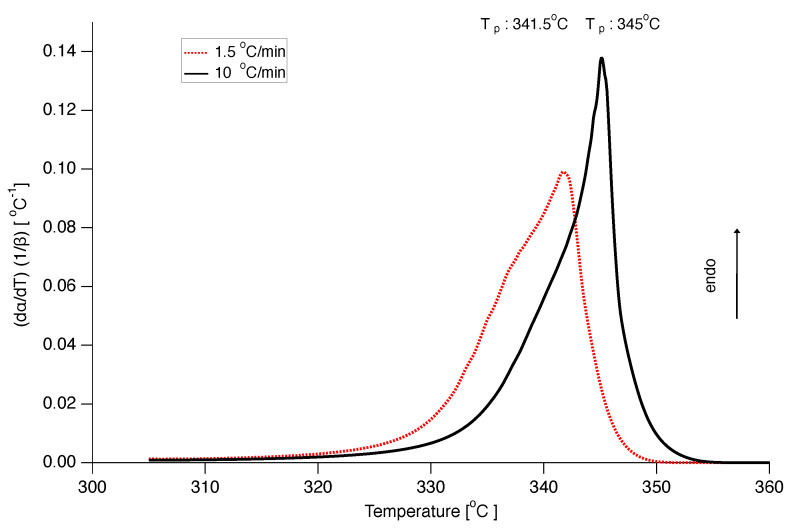
Prediction of differential scanning calorimetry (DSC) curves normalized by heating rate for heating rates 10 ${}^{\circ }$C/min (black solid line) and 1.5 ${}^{\circ }$C/min (red dotted line), using the $A_{1/2}$ model with the kinetic parameters calculated through the isoconversional analysis.

The $A_{1/2}$ model with the experimentally determined $E_{\alpha }$ and $A_{\alpha }$ was also used to reconstruct DSC curves at different heating rates. The results are depicted in Figure 15. Although the shape of the calculated DSC curves is somewhat off, the (apparent) peak melting temperature as a function of heating rate is in good agreement with the experimental values.

**Figure 16:**
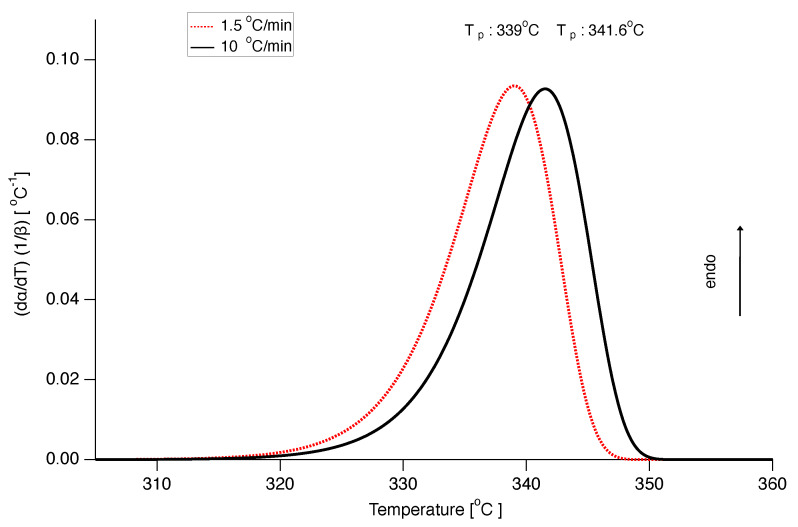
Prediction of DSC curves normalized by heating rate for heating rates 10 ${}^{\circ }$C/min (black solid line) and 1.5 ${}^{\circ }$C/min (red dotted line) using the $A_{1/3}$ model with constant *E* and ln(A).

Also, the $A_{1/3}$ model with constant *E* and ln(A) parameters was used to construct DSC curves with similar good results as for the $A_{1/2}$ model, as can be seen in Figure 16, although the peak melting temperature at a heating rate of 10 ${}^{\circ }$C/min is somewhat too low.

It follows that both the $A_{1/2}$ with conversion-dependent kinetic parameters and the $A_{1/3}$ model with constant *E* and ln(A) are able to describe both isothermal experiments and constant heating-rate DSC curves. Nevertheless, it appears that the fit of both the isothermal and constant heating rate experiments with the $A_{1/2}$ model is slightly better.

A reason could be that the isothermal experiments, that are best fitted with the $A_{1/3}$ model mainly probe the melting behaviour at long times and relatively low temperatures, whereas the rate-dependent DSC measurements that are the input for the isoconversional analysis leading to the $A_{1/2}$ model, are determined by the melting response at relatively short times and high temperatures. Another reason could be that the nucleation and growth processes that underlie the Avrami-Erofeev equations have rate constants with different temperature dependencies [38], of which the combined effect on conversion is more pronounced in the constant heating experiments that cover a relatively large temperature range, compared to the isothermal experiments that were only performed in the narrow temperature range from 332 ${}^{\circ }$C to 337 ${}^{\circ }$C. It is well-known that an overall rate constant with contributions from several underlying processes typically leads to an apparent activation energy that depends on conversion [39].

It is difficult to interpret the experimentally found reaction model $A_{1/2}$, as both the activation energy and the pre-exponential factor are not constant. However, in principle, both the $A_{1/2}$ and $A_{1/3}$ models relate to nucleation with pre-existing nuclei [40,41,42], in agreement with the suggestion by Toda et al. [7,8] of nucleation-driven kinetics in polymer melting.

## 4. Conclusions

It was shown that the melting of nascent PTFE powder exhibits pronounced kinetic effects. This could be of importance for industrial applications, such as the production of PTFE membranes, where part of the nascent material is molten by annealing between the melting temperature of the melt-crystallised material and the first apparent melting temperature, a process which is sometimes referred to as “amorphous interlocking” [43]. During annealing in this temperature range, slow melting will occur, gradually increasing the amount of amorphous material that remains present upon cooling. Friedman’s isoconversional method was applied to DSC heating curves with different heating rates in order to determine an apparent activation energy as a function of conversion for the melting of nascent PTFE. By determination of the kinetic triplet through application of the compensation effect, it was found that a nucleation and growth mechanism, the so-called Avrami-Erofeev model with n=1/2 ($A_{1/2}$ model), can be used to describe the kinetics of the rate-dependent melting of nascent PTFE powser. In addition, isothermal melting experiments were performed in the temperature range of $327{\;}^{\circ }C<T<344{\;}^{\circ }$C, where delayed melting of PTFE was observed, with complete melting only occurring after up to several hours. Also these isothermal experiments were well described by the $A_{1/2}$ model.

Interestingly, it was found that the both the isothermal and constant heating rate experiments could also be described with an Avrami-Erofeev model with n=1/3 ($A_{1/3}$ model) with a *constant* activation energy and pre-exponential factor, although not quite as good as with the $A_{1/2}$ model. The use of Avrami-Erofeev models to describe the melting kinetics of nascent PTFE, would be in agreement with nucleation-driven kinetics in polymer melting as proposed by Toda et al. [7,8].

## Figures and Tables

**Table 1:** Algebraic expressions for f(a) function of the common mechanisms operating in solid-state reactions.

Number	Model	f(α)	Rate-Determining Mechanism
1	$F_{1/3}$	$\left(3/2\right){\left(1-\alpha \right)}^{1/3}$	Chemical reaction
2	$F_{3/4}$	$4{\left(1-\alpha \right)}^{3/4}$	Chemical reaction
3	$F_{3/2}$	$2{\left(1-\alpha \right)}^{3/2}$	Chemical reaction
4	$F_{2}$	${\left(1-\alpha \right)}^{2}$	Chemical reaction
5	$F_{3}$	$\left(1/2\right){\left(1-\alpha \right)}^{3}$	Chemical reaction
6	$F_{4}$	$\left(1/3\right){\left(1-\alpha \right)}^{4}$	Chemical reaction
7	$G_{1}$	1/[2(1-α)]	Chemical reaction
8	$G_{2}$	$1/[3{\left(1-\alpha \right)}^{2}]$	Chemical reaction
9	$G_{3}$	$1/[4{\left(1-\alpha \right)}^{3}]$	Chemical reaction
10	$P_{3/2}$	$\left(2/3\right)\alpha^{-1/2}$	Nucleation (power law)
11	$P_{1/2}$	$2\alpha^{1/2}$	Nucleation (power law)
12	$P_{1/3}$	$3\alpha^{2/3}$	Nucleation (power law)
13	$P_{1/4}$	$4\alpha^{3/4}$	Nucleation (power law)
14	$P_{2}$	$\left(1/2\right)\alpha^{-1}$	Nucleation (parabolic law)
15	$E_{1}$	α	Nucleation (exponential law)
16	$E_{2}$	α/2	Nucleation (exponential law)
17	$A_{1},F_{1}$	1-α	Random nucleation/ first order (Mampel)
18	$A_{2/3}$	$\left(2/3\right)\left(1-\alpha \right){\left[-\ln\left(1-\alpha \right)\right]}^{-1/2}$	Random nucleation (Avrami-Erofeev)
19	$A_{3/2}$	$\left(3/2\right)\left(1-\alpha \right){\left[-\ln\left(1-\alpha \right)\right]}^{1/3}$	Random nucleation (Avrami-Erofeev)
20	$A_{3/4}$	$\left(3/4\right)\left(1-\alpha \right){\left[-\ln\left(1-\alpha \right)\right]}^{-1/3}$	Random nucleation (Avrami-Erofeev)
21	$A_{5/2}$	$\left(5/2\right)\left(1-\alpha \right){\left[-\ln\left(1-\alpha \right)\right]}^{3/5}$	Random nucleation (Avrami-Erofeev)
22	$A_{2}$	$2\left(1-\alpha \right){\left[-\ln\left(1-\alpha \right)\right]}^{1/2}$	Random nucleation (Avrami-Erofeev)
23	$A_{3}$	$3\left(1-\alpha \right){\left[-\ln\left(1-\alpha \right)\right]}^{2/3}$	Random nucleation (Avrami-Erofeev)
24	$A_{4}$	$4\left(1-\alpha \right){\left[-\ln\left(1-\alpha \right)\right]}^{3/4}$	Random nucleation (Avrami-Erofeev)
25	$A_{1/2}$	$\left(1/2\right)\left(1-\alpha \right){\left[-\ln\left(1-\alpha \right)\right]}^{-1}$	Random nucleation (Avrami-Erofeev)
26	$A_{1/3}$	$\left(1/3\right)\left(1-\alpha \right){\left[-\ln\left(1-\alpha \right)\right]}^{-2}$	Random nucleation (Avrami-Erofeev)
27	$A_{1/4}$	$\left(1/4\right)\left(1-\alpha \right){\left[-\ln\left(1-\alpha \right)\right]}^{-3}$	Random nucleation (Avrami-Erofeev)
28	$R_{1},F_{0},P_{1}$	1	Contracting disc
29	$R_{2},F_{1/2}$	$2{\left(1-\alpha \right)}^{2/3}$	Contracting cylinder
30	$R_{3},F_{2/3}$	$3{\left(1-\alpha \right)}^{2/3}$	Contracting sphere
31	$D_{1}$	1/(2α)	One-dimensional diffusion
32	$D_{2}$	$-{\left[\ln\left(1-\alpha \right)\right]}^{-1}$	Three-dimensional diffusion
33	$D_{3}$	$\left(3/2\right){\left(1-\alpha \right)}^{2/3}{\left[1-{\left(1-\alpha \right)}^{1/3}\right]}^{-1}$	Three-dimensional diffusion (Jander)
34	$D_{4}$	$\left(3/2\right){\left[{\left(1-\alpha \right)}^{-1/3}-1\right]}^{-1}$	Three-dimensional diffusion (Ginstling-Brounshtein)

**Figure A1:**
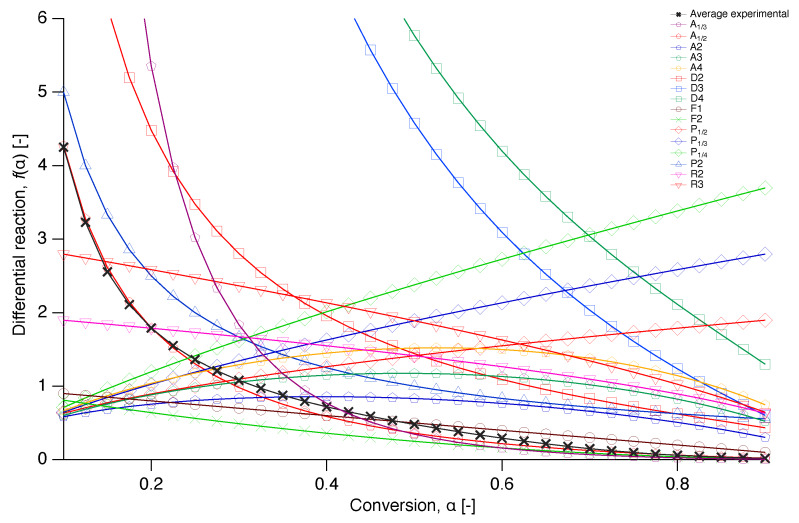
Plots of the differential reaction against the conversion for selected models together with the numerically reconstructed differential reaction of the average value at each conversion of the experimental data.
